# Cross-talk of renal cells through WNT signal transduction in the development of fibrotic kidneys

**DOI:** 10.3389/fcell.2024.1517181

**Published:** 2025-02-12

**Authors:** Yuhong Chen, Chao Xue

**Affiliations:** Department of Nephrology, The Second Affiliated Hospital of Guangxi Medical University, Nanning, China

**Keywords:** cross-talk, kidney fibrosis, wnt/β-catenin signaling, macrophage, fibroblast

## Abstract

Chronic kidney disease (CKD) is a progressive condition that can lead to chronic renal failure (CRF), affecting 8%–16% of adults globally and imposing a significant burden on healthcare systems. Renal fibrosis is a key pathological hallmark of CKD progression and is linked to poor prognosis. Multiple signaling pathways, including WNT/β-catenin.Aberrant activation of WNT/β-catenin is implicated in renal fibrosis. The roles of renal macrophages and fibroblasts are pivotal in fibrosis progression and prognosis.

## Introduction

Chronic kidney disease (CKD) has been demonstrated to be a progressive disease that can gradually advance to chronic renal failure (CRF) (Bikbov et al., 2020). Research indicates that 8%–16% of the adult population globally is affected by CKD, and its high prevalence and cost of treatment impose a substantial burden on global healthcare systems (Bikbov et al., 2020; Chen et al., 2019). Renal fibrosis represents a pivotal pathological feature of CKD progression (Chen et al., 2019), and fibrosis is associated with disease progression and poor prognosis (Ruiz-Ortega et al., 2020; Breyer and Susztak, 2016).

Kidney fibrosis is a complex and irreversible pathology involving the activation of multiple signaling pathways, including the WNT/β-catenin signaling pathway, the TGF-β-Smad signaling pathway, and the Notch pathway (Liu, 2011; Ding et al., 2014; Murea et al., 2010). Among these, the WNT/β-catenin signaling pathway plays a significant role in the process of renal fibrosis. WNT signaling is a key regulator in renal development, regulating cell proliferation, differentiation, and tissue patterning, thereby directing normal renal development (Little and McMahon, 2012). WNT/β-catenin signaling is relatively silent in normal adult kidney; however, it is aberrantly activated in various diseases of the renal, and is implicated in diverse renal pathologies, including renal fibrosis and acute kidney injury (AKI) in CKD (He et al., 2009; Luo et al., 2018).

Despite significant advances in understanding the regulation of WNT/β-catenin pathway expression and its mechanism of action in renal injury have been greatly discovered and elaborated in recent years, the kidney remains a functionally highly differentiated organ, comprising a diverse range of immune cells. Furthermore, renal fibrosis is a progressive disease that is accompanied by the infiltration of a variety of immune cells during its development. The role and function of macrophages undergo significant changes, especially. These changes not only affect the progression of renal fibrosis, but also determine the severity and prognosis of the disease. The role of renal macrophages and fibroblasts in renal fibrosis is discussed in the following section.

### WNT signal composition

The WNT signaling pathways comprise both canonical and non-canonical signaling pathways. The canonical WNT signaling pathway is also known as the WNT/β-catenin pathway. The non-canonical WNT signaling pathways include the WNT/Ca^2^⁺ pathway and the WNT/PCP pathway also known as the WNT/c-JNK signaling pathway (Chae and Bothwell, 2018). From lower organisms to higher organisms, the WNT signaling pathway has retained a high degree of conservation across species. Accumulating evidence indicates that the WNT signaling pathway plays important roles in both disease and health. The canonical WNT signaling pathway exerts significant influence over cell proliferation, differentiation and polarisation during embryonic development. It plays a key role in maintaining tissue homeostasis, stem cell differentiation and self-renewal, and is linked to cancer development. The non-canonical WNT signaling pathway is instrumental for regulating cell polarity, shape and migration, and plays a pivotal role in tissue pattern formation and cell migration during embryonic development (Nusse and Clevers, 2017; Miao et al., 2020; Clevers and Nusse, 2012; Liu et al., 2022; Dapkunas et al., 2019).

### The role of β-catenin-dependent signaling in kidney fibrosis

WNT/β-catenin signaling is relatively quiescent in normal adult kidneys. However, aberrant activation of WNT/β-catenin signaling has been observed in renal injury, particularly within the renal tubules (Luo et al., 2018; DiRocco et al., 2013) (Table 1). In mice with tubular β-catenin deletion, the renal function remained normal in the steady state. Conversely, the induction of AKI following the specific knockdown of β-catenin revealed that the absence of endogenous β-catenin exacerbates renal tubular injury and acute renal failure, while also promoting promotes renal tubular cell apoptosis. Under this condition, the mice that had undergone IRI/AKI exhibited more pronounced renal damage.

**TABLE 1:** The role of WNT/β-catenin signaling in various kidney diseases and renal injury repair.

Stage	Models/Species	WNT/β-catenin pathway contributes to the development of renal fibrosis	Role	References
AKI to CKD	Mouse	Without inflammation and epithelial injury, epithelial-derived Wnt1 directly acts on stromal myofibroblast progenitor cells, thereby driving classical Wnt signaling and fibrosis, leading to the transition from AKI to maladaptive repair and CKD.	Detrimental	Maarouf, O. H., et al. (2016). Paracrine Wnt1 drives interstitial fibrosis without inflammation by tubulointerstitial cross-talk. *Journal of the American Society of Nephrology*, 27 (3), 781–790. https://doi.org/10.1681/ASN.2014121188
	Mouse	Transient activation of the Wnt/β-catenin signaling pathway promotes kidney repair following AKI.	Transient: protective	Li, H., et al. (2022). Tubular β-catenin alleviates mitochondrial dysfunction and cell death in acute kidney injury. *Cell Death & Disease*, 13 (12), 1,050. https://doi.org/10.1038/s41419-022-05395-3
	Mouse		Transient: protective	Zhou, D., et al. (2012). Tubule-specific ablation of endogenous β-catenin aggravates acute kidney injury in mice. *Kidney International*, 82 (5), 537–547. https://doi.org/10.1038/ki.2012.173
	Mouse	Persistent and uncontrolled activation of the Wnt/β-catenin signaling pathway promotes the progression from AKI to CKD.	Sustained: detrimental	Xiao, L., et al. (2015). Sustained activation of Wnt/β-catenin signaling drives AKI to CKD progression. *Journal of the American Society of Nephrology*, 26 (7), 1746–1758. https://doi.org/10.1681/ASN.2015040449
CKD	Mouse (age-related renal fibrosis)	Endogenous Wnt/β-catenin activity antagonists, Klotho and Dickkopf-1 (DKK1), eliminate renal fibrosis in mice	Detrimental	Miao, J., et al. (2019). Wnt/β-catenin/RAS signaling mediates age-related renal fibrosis and is associated with mitochondrial dysfunction. *Aging Cell*, 18 (1), e13004. https://doi.org/10.1111/acel.13004
	Human (HKC-8)	The small molecule inhibitor ICG-001 blocks Wnt/β-catenin signaling, thereby hindering AOPP-induced EMT (epithelial-mesenchymal transition)	Detrimental	Feng, H., et al. (2020). AGE receptor 1 silencing enhances advanced oxidative protein product-induced epithelial-to-mesenchymal transition of human kidney proximal tubular epithelial cells via RAGE activation. *Biochemical and Biophysical Research Communications*, 529 (3), 735–741. https://doi.org/10.1016/j.bbrc.2020.06.144
	Mouse	Blocking Wnt secretion by gene deletion of Wls in renal tubules significantly inhibits myofibroblast activation and reduces renal fibrosis following unilateral ureteral obstruction. Blocking Wnt secretion in renal tubules (rather than in fibroblasts) suppresses fibroblast activation and reduces renal fibrosis following injury	Detrimental	Zhou, D., et al. (2017). Tubule-derived Wnts are required for fibroblast activation and kidney fibrosis. *Journal of the American Society of Nephrology*, 28 (4), 944–957. https://doi.org/10.1681/ASN.2016080902
	Human (IgAN/FSGS MN)	Biopsy specimens from CKD patients show increased expression of multiple Wnt proteins, primarily in the renal tubular epithelium		
	Human (MCN/IgAN/DN/MN/LN)	In various human CKD conditions, upregulation of WNT9a has been observed	Detrimental	Luo, C., et al. (2018). Wnt9a promotes renal fibrosis by accelerating cellular senescence in tubular epithelial cells. *Journal of the American Society of Nephrology*, 29 (4), 1,069–1,083. https://doi.org/10.1681/ASN.2017050574
	Human (LN)	The classical WNT/β-catenin signaling pathway is activated in patients with lupus nephritis	Detrimental	Wang, X.-D., et al. (2013). Aberrant activation of the WNT/β-catenin signaling pathway in lupus nephritis. *PLoS ONE*, 8 (3), e84852. https://doi.org/10.1371/journal.pone.0084852
	Human (DN/FSGS)	In human proteinuric nephropathy, upregulation of Wnt1 and active β-catenin has been observed in podocytes	Detrimental	Dai, C., et al. (2009). Wnt/β-catenin signaling promotes podocyte dysfunction and albuminuria. *Journal of the American Society of Nephrology*, 20 (9), 1997–2008. https://doi.org/10.1681/ASN.2009010019

Some of the subjects died as a result. The stable activation of β-catenin in the tubule of AKI mice has been demonstrated to reduce the apoptosis and necroptosis of renal tubular cell caused by AKI, thereby restoring mitochondrial homeostasis.

This is achieved by increasing mitochondrial biosynthesis and restoring mitochondrial quality (Li et al., 2022a). In IRI/AKI kidneys, knockdown of WNT7B resulted in a reduced response of the canonical WNT pathway and a significant reduction in the kidney’s ability to repair damage. Conversely, renal repair was enhanced by injection of WNT pathway agonists (Lin et al., 2010). This suggests that the transient activation of renal β-catenin in the setting of AKI is a protective response that reduces apoptosis and enhances proximal tubular proliferation, facilitating tissue repair and cell regeneration. In light of these findings Tian et al. (2020). proposed that sequential combination therapy comprising an initial treatment with a WNT agonist, followed by a WNT antagonist treatment, could prove an effective strategy for reducing the risk of death and fibrosis associated with AKI. On the other side, prolonged β-catenin-dependent signalling activation has been linked to renal fibrosis development.

**FIGURE 1:**
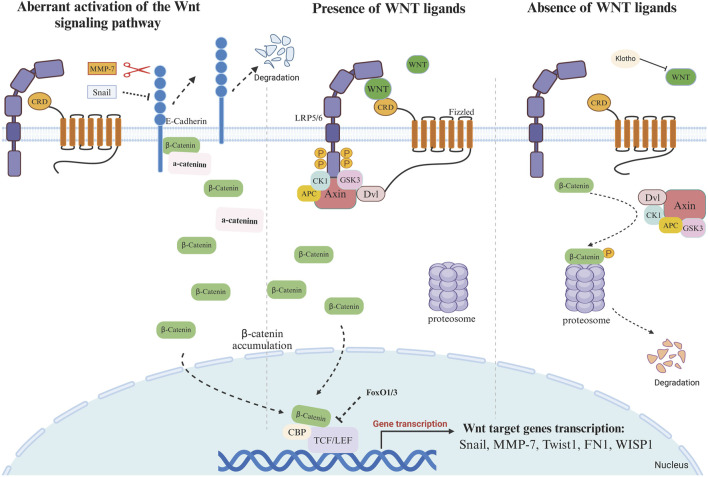
Activation or silencing of WNT signaling pathway in the presence or absence of WNT ligands 1. Absence of Wnt ligand: In the absence of WNT ligands, the “destruction complex” continues to function and binds to cytoplasmic β-catenin. The rupture complex is mainly composed of Axin, APC, CK1, and GSK3β. The main role of this complex is to phosphorylate cytoplasmic β-catenin, thereby promoting its degradation. In the “destruction complex”, Axin acts as a scaffolding protein, responsible for assembling other proteins. APC regulates the phosphorylation and binding of β-catenin, and GSK3β and CK1 phosphorylate β-catenin. Phosphorylated β-catenin is ubiquitinated and then degraded by the proteasome. The continuous degradation of β-catenin leads to the inability of β-catenin to accumulate in the cytoplasm, preventing it from entering the nucleus to bind to the TCF/LEF transcription factor. In the absence of β-catenin, Groucho binds to TCF/LEF and hinders the transcriptional activation of genes, thus inhibiting the expression of Wnt target genes. FoxO1 and FoxO3 can competitively bind to β-catenin, and this binding prevents the binding of β-catenin to TCF/LEF, thus affecting the downstream of Wnt signaling. Klotho can bind to Wnt (not β-catenin) to block Wnt-triggered β-catenin activation and nuclear translocation. 2. Presence of Wnt signal ligand Binding of Wnt ligands to Frizzled (Fz) and co-receptors LRP5/6 on the cell membrane surface activates Dishevelled (Dsh/DVL) proteins, which inhibit the activity of the “destruction complex”. Axin binds to phosphorylated LRP5/6 and forms a signalling complex that prevents the assembly of the β-catenin destruction complex. GSK3β activity is inhibited, and β-catenin is no longer phosphorylated and degraded, thus accumulating in the cytoplasm. The stabilised β-catenin gradually accumulates and is transferred to the nucleus, where β-catenin binds to TCF/LEF transcription factors to form a transcriptional activation complex, which initiates the expression of Wnt target genes, such as Snail, MMP-7, Twistl, FN1, WISPI, c-Myc, Cyclin D1, Axin2 and LEF1.3 Aberrant activation of the Wnt signaling pathway Aberrant activation of Snail1 inhibits the expression of E-cadherin, and MMP-7, with its proteolytic activity, is able to degrade E-cadherin. E-cadherin and β-catenin form a complex on the cell membrane to maintain the membrane localization of β-catenin. When E-cadherin is degraded, β-catenin, which was originally bound to E-cadherin, is released and accumulates in the cytoplasm. β-Catenin translocates into the nucleus and binds to TCF/LEF transcription factor to initiate the expression of Wnt target gene.

This is due to the fact that severe IRI results in prolonged and uncontrolled activation of the Wnt/β-catenin pathway, which in turn leads to the development of kidney fibrosis. This is characterized by the activation of interstitial myofibroblasts and the excessive deposition of extracellular matrix deposition (Zhou et al., 2012; Xiao et al., 2016). FoxO1/3 transcription factors compete with TCF/LEF for binding to β-catenin, leading to a shift in signaling from TCF/LEF-dependent pathways to FoxO3-dependent pathways (Figure 1). This inhibits TCF/LEF-dependent transcription and prevents renal tubular epithelial cell (TEC) apoptosis. Accordingly Nlandu-Khodo et al. (2020) propose that the activation of the β-catenin-dependent signaling pathway in the proximal renal tubule exerts antifibrotic and renal function-protective effects during both periods of unilateral ischemia-reperfusion and aristolochic acid-induced AKI to CKD models, contingent on the presence of FoxO3 transcription factors *in vivo*.

The WNT signaling pathway promotes renal fibrosis by activating the β-catenin signaling pathway, which subsequently drives the process of epithelial-mesenchymal transition (EMT). Partial epithelial-mesenchymal transition (p-EMT) refers to a transitional phenotype in which renal tubular epithelial cells (TECs), under specific stimuli, partially lose their epithelial characteristics without fully acquiring mesenchymal cell traits (Sheng and Zhuang, 2020). Unlike complete EMT, p-EMT cells retain some epithelial features, such as the expression of E-cadherin, while simultaneously expressing certain mesenchymal markers, such as α-SMA or fibronectin. This hybrid phenotype is widely observed in kidney fibrosis and represents a dynamic response of TECs to signals from a damaging microenvironment. Furthermore, fibrotic responses activated through EMT may not require epithelial cells to fully transition into myofibroblasts. Instead, p-EMT cells acquire the ability to produce pro-fibrotic cytokines and growth factors. The p-EMT process is sufficient to induce TEC dysfunction by triggering cell cycle arrest and inflammation, which, in turn, promotes the secretion of pro-fibrotic factors (Lovisa et al., 2015). This cascade facilitates fibroblast activation and exacerbates kidney fibrosis. p-EMT is an indispensable stage in the development of fibrosis. By intervening in the EMT signaling pathways, particularly the Wnt/β-catenin pathway, potential strategies for treating kidney fibrosis can be developed (Hu et al., 2022).

During renal fibrosis, the activation of the WNT signaling pathway has been observed to promote the expression of Snail1 (a member of the zinc finger family of transcription factors), which remains silent throughout the course of kidney development (Figure 1). Aberrant activation of Snail1 directly inhibits the expression of E-cadherin, which blocks and disrupts adhesion between epithelial cells and triggers EMT, thereby initiating a vicious cycle of fibroblast activation and ECM deposition, which exacerbates the fibrosis process. During renal fibrosis, Snai1 not only acts as a target of WNT/β-catenin to regulate the EMT process, but also cooperates with WNT ligands to induce signaling. Snail1 can induce renal tubular epithelial cells (TECs) to acquire a partial epithelial-mesenchymal transition (p-EMT) phenotype. The secretion of fibrosis-related cytokines and growth factors can further activate surrounding stromal cells and immune cells, creating a feedback loop that promotes fibrosis. Modulating p-EMT, rather than complete EMT, may be an effective strategy for treating kidney fibrosis (Grande et al., 2015).

Decreased expression of E-cadherin leads to release of β-catenin from adherens junctions and its subsequent translocation to the nucleus (Schunk et al., 2021; Boutet et al., 2006; Wang et al., 2010). Conversely, conditional knockout of Snai1 inhibiting the EMT process maintains the integrity of renal tubular epithelial cells and reduces immune cell infiltration in renal fibrosis, while restoring proliferation, dedifferentiation-related repair and regeneration of the renal parenchyma and thereby reducing interstitial fibrosis (Lovisa et al., 2015; Qi et al., 2021) (Figure 1). Expression of RSPO1 is increased in the kidney of obese mice induced by high-fat diet. Knockdown of RSPO1 attenuated renal injury and fibrosis. By binding to LGR4, RSPO1 enhanced β-catenin activity and redistribution in renal tubular cells. This activation of the Wnt/β-catenin signaling pathway promotes the EMT process (Su et al., 2021).

### WNT and macrophage

**FIGURE 2:**
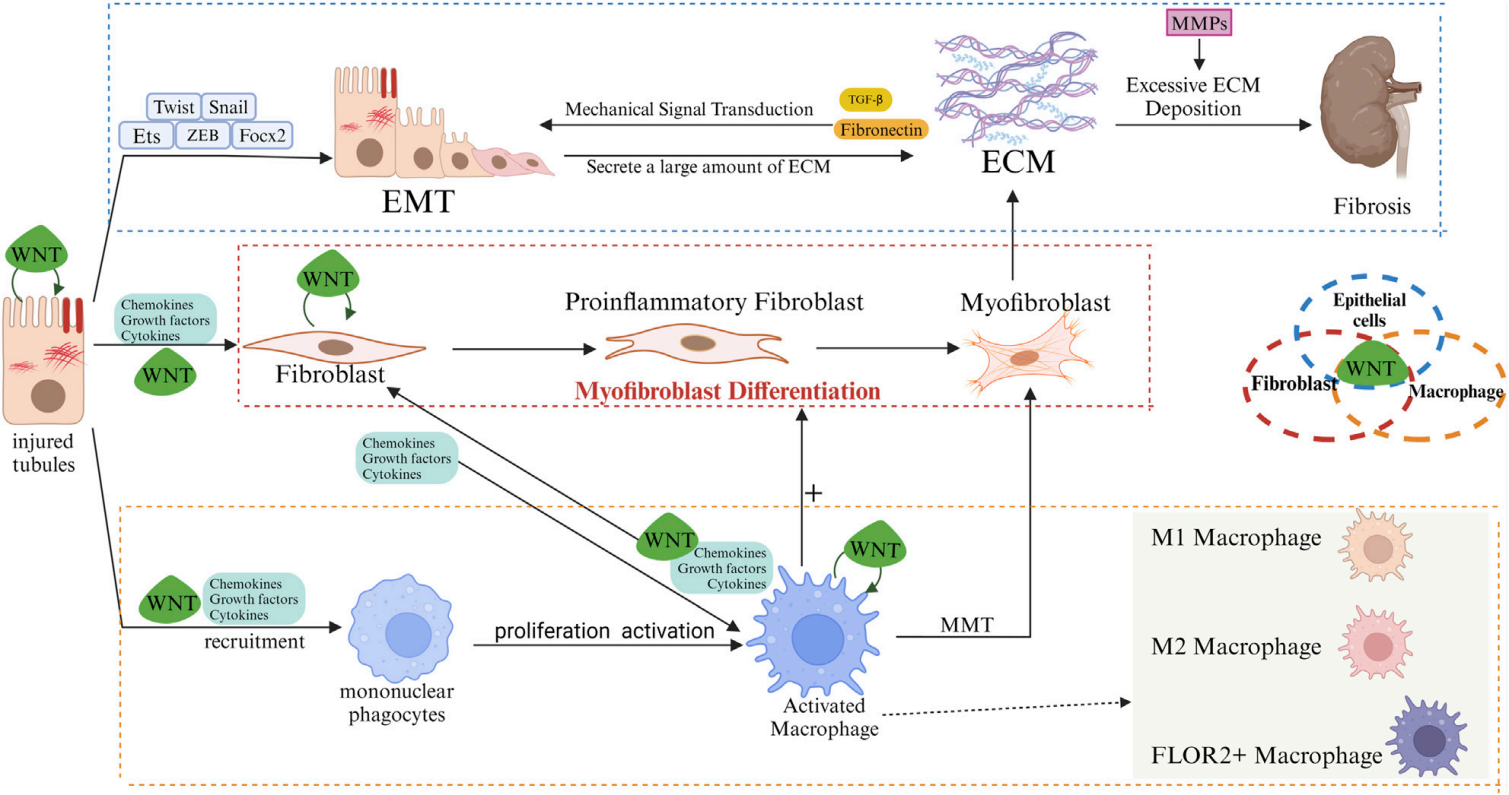
Role of WNT/β-catenin in regulating interactions of renal tubular epithelia/fibroblasts/macrophages. In the early stages of fibrosis, injured renal tubular epithelial cells release pro-inflammatory mediators and chemokines, among others, to recruit and activate macrophages. Macrophages undergo M1 polarization, and M1 macrophages clear pathogens while secreting large amounts of pro-inflammatory factors, exacerbating tubular and interstitial damage. In the later stages of fibrosis, M2 macrophages (alternatively activated macrophages) gradually replace M1 macrophages. M2 macrophages promote the proliferation and activation of fibroblasts into myofibroblasts by secreting pro-fibrotic factors such as TGF-β and PDGF. Myofibroblasts synthesize large amounts of ECM, including collagen, fibronectin and hyaluronic acid. Fibroblasts promote macrophage activation, and activated macrophages can become myofibroblasts (MMT). The population of inflammatory fibroblasts is part of the intermediate state. In the process of differentiation from fibroblasts to myofibroblasts. Inflammatory fibroblast populations attract macrophages in the early stages of fibrosis and induce monocytes to differentiate into FOLR2+ macrophages. Subsequently, these FOLR2+ macrophages induce the transformation of pro-inflammatory fibroblast populations into myofibroblasts via the WNT/β-catenin pathway. Excessive ECM deposition disrupts the normal structure of renal tissue, leading to progressive irreversibility of fibrosis. Myofibroblasts can also originate from activated macrophages (MMT). Injured renal tubular epithelial cells undergo EMT, and Snail, Twist, ZEB, FOXC, and Ets are important transcription factors in the process of EMT. During EMT, renal tubular epithelial cells lose their polarity and tight junctions, and are gradually transformed into mesenchymal cells with fibroblast-like features, expressing mesenchymal markers such as α-SMA, and producing ECM components (especially Fibronectin) bind to Integrins in the tubular epithelium, altering cell adhesion properties and further inducing EMT. Meanwhile, the mechanical stress of ECM, which increases during fibrosis, can activate mechanosensory signaling pathways in the epithelium, which can also transmit signals via Integrins for the development of EMT. In normal tissue repair, MMPs maintain the dynamic balance of the ECM to promote tissue repair and regeneration by removing damaged tissue and degrading ECM components. But MMPs are over-activated in fibrotic environments, and MMPs not only degrade the basement membrane, making it easier for cells to migrate and facilitating the EMT process, but also participate in the degradation and remodelling of the ECM, which provides space for fibrotic tissue to expand, and release some pro-fibrotic cytokines that are bound and stored by the ECM, such as TGF-β, which further facilitates the activation of fibroblasts and ECM deposition, leading to increased fibrosis. Epithelial cells can autocrine WNT ligand in a fibrotic environment, activating WNT/β-catenin signaling to generate EMT, which allows epithelial cells to progressively lose epithelial properties (cell polarity and tight junctions) and to acquire fibroblastic properties. Paracrine WNT ligand activates neighbouring fibroblasts to promote their proliferation and activation into myofibroblasts, resulting in excessive ECM deposition. Paracrine WNT ligands also modulate the behaviour of macrophages, which undergo M2 polarisation and release pro-fibrotic factors. In fibroblasts, the maintenance of their activated state and pro-fibrotic phenotype is helped by autocrine and paracrine WNT. Activation of the WNT pathway induces pro-inflammatory activation of macrophages and polarization to the M2 phenotype, whereas macrophage-derived WNT affects fibroblast activation and proliferation. Similarly, renal tubular cells can directly interact with WNT ligands derived from macrophages and fibroblasts.

In the event of kidney injury occurs, macrophages undergo a phenotypic shift and release multiple WNT ligands, which act on epithelial and mesenchymal cells to induce repair and tissue regeneration (Lin et al., 2010). The secretion of the WNT ligands by renal tubules in a fibrotic environment can significantly has been demonstrated to exert a significant influence on the activity and function of macrophages. Furthermore, activated macrophages have been observed to secrete a diverse range of cytokines, which in turn stimulate the proliferation and activation of fibroblasts. This process ultimately leads to the production of a substantial quantity collagen and other extracellular matrix components by the fibroblasts, resulting in the hyperplasia and exacerbation of renal interstitial fibrosis. The over-activation of fibroblasts further promotes the recruitment and activation of macrophages, thereby forming a vicious circle (Figure 2).

### WNT ligands mediate macrophage activation and polarization

WNT signaling plays a pivotal role in the regulation of organ fibrosis in inflammatory monocytes and tissue-resident macrophages. Activation of WNT3a/β-catenin signaling pathway has been observed to induce phosphorylation and nuclear translocation of STAT3, which in turn has been demonstrated to aggravate macrophages M2 polarization and promote the process of renal fibrosis. On the other hand, the deletion of β-catenin in macrophages reduces macrophage M2 polarization and diminishes renal fibrosis (Feng et al., 2018).

WNT ligands have been induced in several types of kidney cells, including tubular epithelial cells, mesenchymal fibroblasts, and fibroblasts (Schunk et al., 2021). Fibroblasts promote the activation of macrophages, and activated macrophages can be transformed into myofibroblasts. This transformation process is also known as MMT (Chen et al., 2022) (Figure 2). Macrophage ablation improves myofibroblast activation and prevents myofibroblast accumulation and tissue fibrosis (Pai et al., 2020). Inflammatory fibroblast populations were found to belong to an intermediate state in the differentiation process from fibroblasts to myofibroblasts using single-cell sequencing technology. In the early stage of fibrosis, the inflammatory fibroblast population attracts macrophages and induces monocytes to differentiate into FOLR2+ macrophages. Subsequently, these FOLR2+ macrophages induce the transformation of pro-inflammatory fibroblast populations into secreting myofibroblasts via activation of the WNT/β-catenin pathway. Inhibition of the β-catenin/TCF interaction or blockade of WNT ligand secretion can block the macrophage-induced transformation of pro-inflammatory fibroblast populations into ECM-secreting myofibroblasts (Cohen et al., 2024) (Figure 2). In cancer and diabetic nephropathy, inflammatory fibroblast populations attract CD14^+^ monocytes and induce their polarization into FOLR2+ macrophages (Fu et al., 2022; Timperi et al., 2022). Yi-Lin Zhang et al. found that extracellular matrix remodeling-associated macrophages (EAM) differentiated from infiltrating monocytes were associated with renal fibrosis, and that macrophages and damaged tubules in the later stages of fibrosis were spatially closer to fibroblasts, whereas renal-resident macrophages mainly behaved in a way that promoted tissue repair (Zhang et al., 2024).

### Macrophage-derived WNT signal and the effect of macrophage on WNT signaling pathway

In the kidney, macrophages are an important source of WNT ligands. Macrophage-derived WNTs induced M2 polarization in resting macrophages while expressing fibrosis-associated proteins such as fibronectin, collagen, and α-SMA (Tian et al., 2024). Macrophage-derived WNT7b can stimulate epithelial responses in damaged kidney tissue and participate in renal or intestinal epithelial repair (Saha et al., 2016), M2-macrophage-derived WNT7a also promotes the differentiation of lung-resident mesenchymal stem cells into myofibroblasts (Hou et al., 2018).Blockade of WNT secretion from mouse macrophages resulted in reduced β-catenin activation, decreased macrophage infiltration and activation, suppressed expression of inflammatory cytokines, inhibited activation and proliferation of mesenchymal fibroblasts, attenuated renal inflammation after obstructive injury, and ameliorated renal fibrotic lesions. The Wnt signaling derived from myeloid cells is a major signaling source during the progression of kidney fibrosis. In the study by Tian et al. (2024), it was found that Wnts derived from macrophages not only activate macrophages themselves but also affect the activation and proliferation of fibroblasts. In mice with myeloid-specific Wls deletion, a significant reduction in β-catenin activation levels in renal tubular epithelial cells was observed, inhibiting β-catenin signaling and kidney fibrosis after ischemic injury.Macrophage-derived Wnts are key factors in activating β-catenin in renal tubules, and these Wnts may trigger β-catenin activation in tubular cells through a paracrine mechanism.

In addition to the secretion of WNT ligands and inflammatory cytokines, activated macrophages also secrete matrix metalloproteinases (MMPs). Macrophage-derived MMP-2, MMP-9, and MMP-12 have been shown to be associated with the progression of renal fibrosis (Tan et al., 2013; La Russa et al., 2024; Baudier et al., 2024). MMPs can directly activate the growth factor TGF-β, which affects signaling and promotes fibroblast activation and proliferation, leading to excessive synthesis of collagen and other ECM components (Wozniak et al., 2021) (Figures 1, 2). Of these MMPs, MMP-7 has been identified as a biomarker for kidney fibrosis (Hirohama et al., 2023). The human MMP-7 gene promoter contains a T-cell factor binding element (TBE) site responsive to the WNT/β-catenin pathway and an activator protein interaction network 1 (AP-1) site required for growth factor activation (He et al., 2012). MMP-7 has the capacity to activate β-catenin through the process of E-cadherin degradation, which is dependent on its proteolytic activity. This causes enhanced nuclear accumulation of β-catenin, which subsequently activates the independent WNT signalling pathway. Furthermore, the activation of β-catenin can facilitate the binding of TCF to the TBE structural domain in the MMP-7 promoter, thereby inducing the trans-activation of the MMP-7 gene and establishing a positive feedback loop (He et al., 2012) (Figure 1). Knockdown of MMP-7 significantly inhibited the expression of fibrosis-related genes: fibronectin and α-SMA, Snail1, and PAI-1, and blocked fibronectin deposition in the kidney (Zhou et al., 2017a).

### WNT and fibroblast

Fibroblasts play a central role in fibrosis after kidney injury. After renal injury, macrophages release soluble mediators, in the presence of which fibroblasts are activated and migrate to the site of injury where they proliferate and synthesize and secrete large quantities of collagen as well as other ECM components such as fibronectin and glycosaminoglycans to repair the damaged tissue. However, excessive activation of fibroblasts and continuous ECM deposition can lead to pathological fibrosis (Huang et al., 2023; Li et al., 2022b). Secretion of WNT ligand by injured renal tubules affects fibroblast behavior. WNT1 ligand serves as a key activator of the canonical WNT signaling pathway (Clevers and Nusse, 2012).Overexpression of WNT1 activates β-catenin signaling by binding to Frizzled receptors on the surface of fibroblasts, inducing activation of renal mesangial fibroblasts, promoting the expression of fibronectin, and accelerating the transition from AKI to CKD. Meanwhile, β-catenin-dependent signaling is crucial for sustaining of αSMA + activity in myofibroblasts after AKI. After removal of WNT ligands, activated fibroblasts readily reverted to a resting phenotype, suggesting that ongoing WNT signaling is necessary for fibroblast activation (Xiao et al., 2016). Activation of fibroblasts and fibrosis were prevented in the absence of WNT ligand in renal tubular epithelial cells, but specific knockdown of WNT ligand in fibroblasts had no significant effect on fibrosis (Zhou et al., 2017b). However, specific ablation of β-catenin in fibroblasts protected the kidney from apoptosis and inflammatory induced by acute ischemia (Zhou et al., 2018a). Maarouf et al. found that proximal tubule-derived WNT1 ligand was enough to trigger renal interstitial fibrosis in the absence of any inflammation and epithelial damage by directly targeting interstitial myofibroblast progenitors through paracrine signaling (Maarouf et al., 2016). Wnt4 activates the Wnt/β-catenin pathway through autocrine signaling and induces mesenchymal fibroblasts to spontaneously differentiate into myofibroblasts (DiRocco et al., 2013).

At the same time, the behavior of fibroblasts is also affected by the Sox9 transcription factor. It was found that SOX9 can act as an “on-off switch” to determine cell behavior during the repair process after kidney injury. SOX9 can promote tissue regeneration and the process of fibrosis, where the duration of regenerative response is a key factor in determining fibrotic healing (Aggarwal et al., 2024; Bugg and Davis, 2024).The upregulation of SOX9 can regulate the expression of cell cycle-related genes to promote the proliferation of fibroblasts, and also interact with MMPs to enhance the migration ability of fibroblasts, thereby promoting the reconstruction of damaged tissues. However, sustained and severe injury caused by prolonged activation of SOX9 leads to the transformation of fibroblasts into myofibroblasts, which in turn promotes collagen synthesis and ECM deposition.

SOX9 is present in myofibroblasts in human and mouse CKD. The cellular state activated by the Sox9 transcription factor is a central hub for the formation and maintenance of myofibroblasts and a major source of sustained, biologically active WNT signaling that drives fibrosis after AKI. Aggarwal et al. (2024) found that after AKI, damaged proximal tubular epithelial cells activate the SOX9 transcription factor, which generates WNT activity to stimulate a fibroproliferative response in neighboring fibroblasts, driving the advancement of AKI to CKD. SOX9 facilitates the activation of fibroblasts and the overdeposition of ECMs by orchestrating the cellular neighborhood of, which promotes the fibrotic process (Bugg and Davis, 2024). In mouse models of pulmonary and hepatic fibrosis, endothelial cells can activate neighboring fibroblasts, which then migrate and deposit stroma in response to SOX9, but endothelial cell-specific Sox9 deficiency reverses these changes (Trogisch et al., 2024). Sox9 deficiency attenuates the injury-induced increase in expression of the fibrosis-associated cytoskeletal-associated factor Nav3 (Raza et al., 2021). SOX9 knockdown significantly inhibited ECM production and granulation tissue proliferation, promoted epithelial regeneration and granulation tissue apoptosis, and attenuated post-injury fibrosis (Morgado-Pascual et al., 2022; Gu et al., 2022).

### Strategies targeting WNT/β-catenin signaling to address renal fibrosis

Recent research have demonstrated that the WNT/β-catenin signaling pathway is crucial in the initiation and progression of renal fibrosis. Abnormal activation of this signaling pathway is closely associated with excessive proliferation of fibroblasts and ECM. Consequently, therapeutic strategies targeting WNT/β-catenin signaling have increasingly become a focus of research (Table 2).

**TABLE 2:** Several drugs or natural compound that target WNT/β-catenin signaling pathway.

Drugs/Natural compounds	Cell/Mouse model	Targeting the WNT/β-catenin pathway mitigate renal fibrosis	References
Resveratrol	UUO TEC	Resveratrol inhibits fibroblast-myofibroblast differentiation (FMD) and epithelial-mesenchymal transition (EMT), reducing kidney fibrosis. Its antifibrotic effect is associated with the suppression of Wnt/β-catenin and other proliferative signaling pathways, and it partially reverses FMD and EMT by inactivating Smad2/3 signaling, thereby inhibiting the myofibroblast phenotype	Zhang, X., et al. (2019). Resveratrol suppresses the myofibroblastic phenotype and fibrosis formation in kidneys via proliferation-related signalling pathways. *British Journal of Pharmacology*, 176 (20), 3845–3858. https://doi.org/10.1111/bph.14842
	RAT (IRI)	Short-term resveratrol intervention improves kidney repair after ischemia and accelerates tubular recovery. It also targets inflammation and pro-fibrotic signals during maladaptive responses, normalizing these processes. Importantly, resveratrol continues to prevent the transition from acute kidney injury to chronic kidney disease even after 5 months of intervention	Martínez-Rojas, M. Á., et al. (2024). A short treatment with resveratrol after a renal ischaemia-reperfusion injury prevents maladaptive repair and long-term chronic kidney disease in rats. *The Journal of Physiology*, 602 (12), 2637–2653. https://doi.org/10.1113/JP285979
API	Mouse (HN)	API improves kidney fibrosis by inhibiting the Wnt/β-catenin pathway. API effectively promotes urinary uric acid (UA) excretion in HN mice and inhibits URAT1 and GLUT.	Li, Y., et al. (2021). Apigenin ameliorates hyperuricemic nephropathy by inhibiting URAT1 and GLUT9 and relieving renal fibrosis via the Wnt/β-catenin pathway. *Phytomedicine*, 85, 153,585. https://doi.org/10.1016/j.phymed.2021.153585
CoQ10	Mouse UUO	CoQ10 inhibits RIP1-RIP3-MLKL-mediated necroinflammatory signaling by suppressing the Wnt3α/β-catenin/GSK-3β pathway in UUO, thereby alleviating renal fibrosis	Jiang, Y. J., et al. (2022). Coenzyme Q10 attenuates renal fibrosis by inhibiting RIP1-RIP3-MLKL-mediated necroinflammation via Wnt3α/β-catenin/GSK-3β signaling in unilateral ureteral obstruction. *International Immunopharmacology*, 107, 108,868. https://doi.org/10.1016/j.intimp.2022.108868
	Human	The decrease of CoQ 10 concentration can promote the progression of renal insufficiency in patients with CKD.	Gvozdjáková, A., et al. (2020). Platelet mitochondrial respiration, endogenous coenzyme Q10, and oxidative stress in patients with chronic kidney disease. *Diagnostics*, 10 (3), 176. https://doi.org/10.3390/diagnostics10030176
Curcumin	Rat (DN)	Curcumin can reduce the accumulation of extracellular matrix in diabetic nephropathy by restoring the downregulation of Wnt/β-catenin signaling	Ho, C., et al. (2016). Curcumin rescues diabetic renal fibrosis by targeting superoxide-mediated Wnt signaling pathways. *American Journal of the Medical Sciences*, 351 (4), 358–365. https://doi.org/10.1016/j.amjms.2015.12.017
Poricoic acid A	Mouse (UUO/adenine-induced CKD)	PAA, as a regulator of tryptophan hydroxylase-1 (TPH-1) expression, alleviates renal fibrosis by modulating the Wnt/β-catenin signaling pathway through its effects on β-catenin protein stability and β-catenin-mediated transcription	Chen, D. Q., et al. (2020). Poricoic acid A as a modulator of TPH-1 expression inhibits renal fibrosis via modulating protein stability of β-catenin and β-catenin-mediated transcription. *Therapeutic Advances in Chronic Disease*, 11, 2040622320962648. https://doi.org/10.1177/2040622320962648
GSK-J4	Mouse (DKD)	GSK-J4 improves kidney dysfunction, glomerulosclerosis, inflammation, and fibrosis in diabetic mice by regulating DKK1	Hung, P.-H., et al. (2022). The histone demethylase inhibitor GSK-J4 is a therapeutic target for the kidney fibrosis of diabetic kidney disease via DKK1 modulation. *International Journal of Molecular Sciences*, 23 (16), 940,907. https://doi.org/10.3390/ijms23169407

Klotho is an anti-aging protein that acts as an antagonist of endogenous WNT/β-catenin signaling. It binds to multiple WNT proteins but not to β-catenin, thereby blocking WNT-induced β-catenin activation and nuclear translocation (Zhou et al., 2019; Xie et al., 2021). In a combined Klotho treatment regimen, the G (2)/M block was bypassed, accompanied by a reduction in fibrocytokine production, inhibition of β-catenin activation and its downstream gene expression, and a decrease in myofibroblast activation and matrix expression (Satoh et al., 2012). Also, targetting zeste homolog 2 enhancer (EZH2) may be a novel therapeutic approach to the amelioration of renal fibrosis subsequent to acute kidney injury. EZH2 is a methyltransferase found upregulated in fibrotic kidneys. Inhibition of EZH2 in renal organoids with the EZH2-specific inhibitor GSK343 blocks SMAD3-dependent cis-complexes and inhibits fibroblast activation (Davis et al., 2022) and transcriptional inactivation of the TGFβ1 signaling pathway (Jiang et al., 2021). Inhibition of EZH2 with 3-dezanepin A (3-DZNeP) can eliminate extracellular matrix protein deposition and α-smooth muscle actin expression in obstructed kidneys (Zhou et al., 2016). Furthermore, it has been shown to reduce the G2/M phase arrest of the renal tubular cell cycle and increase E-cadherin expression. 3-DZNeP has been demonstrated to decrease vimentin expression, attenuate aberrant secretion of pro-fibrotic and pro-inflammatory factors, and block M2 macrophage polarization. Additionally, it has been shown to dephosphorylate AKT and β-catenin *in vivo* and *in vitro*. Notably, it effectively eliminating β-catenin phosphorylation without affecting the expression level of total β-catenin (Zhou et al., 2023). 3-DZNeP has also been observed to inhibit the expression of Snail-1 and Twist (Zhou et al., 2018b).

## Conclusion

Wnt signaling is essential for regulating embryonic development and adult tissue homeostasis. Dysregulation of the Wnt/β-catenin pathway is strongly associated with various diseases, including cancer (Flanagan et al., 2019) and neurodegenerative disorders (Ramakrishna et al., 2023). It is a key driver in cancers such as colorectal cancer, influencing EMT, MET, angiogenesis, fibroblast activation, and tumor metastasis (Yang et al., 2020). Excessive inhibition of Wnt signaling may disrupt tissue homeostasis, impair epithelial repair, and exacerbate neurodegenerative processes like neuron loss and synaptic dysfunction (Serafino et al., 2020; Song et al., 2021). Therefore, while targeting this pathway holds therapeutic potential for kidney fibrosis and chronic kidney disease, careful regulation is crucial to avoid adverse effects, such as increased risks of Alzheimer’s disease. Future studies should prioritize tissue-specific and temporally precise modulation to minimize off-target effects.

This paper provides a concise overview of the recent findings of WNT/β-catenin signalling pathway and its relevant crosstalk between epithelial cells from the injured tubules, the proximal macrophages and fibroblasts in the context of renal fibrosis, offering insights that could enhance the treatment of renal fibrosis. Developing drugs to target the Wnt/β-catenin pathway is a promising approach for treating renal fibrosis. However, further precision is required regarding the temporal and spatial effects of Wnt signalling pathway in the event of kidney injury, given that the development of fibrosis is often accompanied by dynamic activation of WNT signalling and a significant heterogeneity of its related cells, including macrophages and other profibrotic cells. In conclusion, the study of Wnt signalling pathway is important and necessary, with the potential to bring further opportunities and possibilities for the study of chronic kidney disease.

## References

[B1] Aggarwal S. Wang Z. Rincon Fernandez Pacheco D. Rinaldi A. Rajewski A. Callemeyn J. (2024). SOX9 switch links regeneration to fibrosis at the single-cell level in mammalian kidneys. Science 383, eadd6371. 10.1126/science.add6371 38386758 PMC11345873

[B2] Baudier R. L. Orlandi P. F. Yang W. Chen H. Y. Bansal N. Blackston J. W. (2024). Matrix metalloproteinase-2 and CKD progression: the chronic renal insufficiency cohort (CRIC) study. Kidney Med. 6, 100850. 10.1016/j.xkme.2024.100850 39131916 PMC11315214

[B3] Bikbov B. (2020). Global, regional, and national burden of chronic kidney disease, 1990-2017: a systematic analysis for the Global Burden of Disease Study 2017. Lancet 395, 709–733. 10.1016/S0140-6736(20)30045-3 32061315 PMC7049905

[B4] Boutet A. De Frutos C. A. Maxwell P. H. Mayol M. J. Romero J. Nieto M. A. (2006). Snail activation disrupts tissue homeostasis and induces fibrosis in the adult kidney. EMBO J. 25, 5603–5613. 10.1038/sj.emboj.7601421 17093497 PMC1679761

[B5] Breyer M. D. Susztak K. (2016). The next generation of therapeutics for chronic kidney disease. Nat. Rev. Drug Discov. 15, 568–588. 10.1038/nrd.2016.67 27230798 PMC5511522

[B6] Bugg D. Davis J. (2024). Sox9-coordinated cellular neighborhoods generate fibrosis. Cell. Stem Cell. 31, 589–590. 10.1016/j.stem.2024.04.009 38701754

[B7] Chae W.-J. Bothwell A. L. M. (2018). Canonical and non-canonical Wnt signaling in immune cells. Trends Immunol. 39, 830–847. 10.1016/j.it.2018.08.006 30213499 PMC7367500

[B8] Chen J. Tang Y. Zhong Y. Wei B. Huang X. R. Tang P. M. K. (2022). P2Y12 inhibitor clopidogrel inhibits renal fibrosis by blocking macrophage-to-myofibroblast transition. Mol. Ther. 30, 3017–3033. 10.1016/j.ymthe.2022.06.019 35791881 PMC9481993

[B9] Chen T. K. Knicely D. H. Grams M. E. (2019). Chronic kidney disease diagnosis and management: a review. Jama 322, 1294–1304. 10.1001/jama.2019.14745 31573641 PMC7015670

[B10] Clevers H. Nusse R. (2012). Wnt/β-catenin signaling and disease. Cell. 149, 1192–1205. 10.1016/j.cell.2012.05.012 22682243

[B11] Cohen C. Mhaidly R. Croizer H. Kieffer Y. Leclere R. Vincent-Salomon A. (2024). WNT-dependent interaction between inflammatory fibroblasts and FOLR2+ macrophages promotes fibrosis in chronic kidney disease. Nat. Commun. 15, 743. 10.1038/s41467-024-44886-z 38272907 PMC10810789

[B12] Dapkunas A. Rantanen V. Gui Y. Lalowski M. Sainio K. Kuure S. (2019). Simple 3D culture of dissociated kidney mesenchyme mimics nephron progenitor niche and facilitates nephrogenesis Wnt-independently. Sci. Rep. 9, 13433. 10.1038/s41598-019-49526-x 31530822 PMC6748995

[B13] Davis J. L. Kennedy C. Clerkin S. Treacy N. J. Dodd T. Moss C. (2022). Single-cell multiomics reveals the complexity of TGFβ signalling to chromatin in iPSC-derived kidney organoids. Commun. Biol. 5, 1301. 10.1038/s42003-022-04264-1 36435939 PMC9701233

[B14] Ding Y. Kim S. l. Lee S. Y. Koo J. K. Wang Z. Choi M. E. (2014). Autophagy regulates TGF-β expression and suppresses kidney fibrosis induced by unilateral ureteral obstruction. J. Am. Soc. Nephrol. 25, 2835–2846. 10.1681/ASN.2013101068 24854279 PMC4243349

[B15] DiRocco D. P. Kobayashi A. Taketo M. M. McMahon A. P. Humphreys B. D. (2013). Wnt4/β-catenin signaling in medullary kidney myofibroblasts. J. Am. Soc. Nephrol. 24, 1399–1412. 10.1681/ASN.2012050512 23766539 PMC3752942

[B16] Feng Y. Ren J. Gui Y. Wei W. Shu B. Lu Q. (2018). Wnt/β-Catenin-Promoted macrophage alternative activation contributes to kidney fibrosis. J. Am. Soc. Nephrol. 29, 182–193. 10.1681/ASN.2017040391 29021383 PMC5748914

[B17] Flanagan D. J. Vincan E. Phesse T. J. (2019). Wnt signaling in cancer: not a binary ON:OFF switch. Cancer Res. 79, 5901–5906. 10.1158/0008-5472.CAN-19-1362 31431458 PMC7616966

[B18] Fu J. Sun Z. Wang X. Zhang T. Yuan W. Salem F. (2022). The single-cell landscape of kidney immune cells reveals transcriptional heterogeneity in early diabetic kidney disease. Kidney Int. 102, 1291–1304. 10.1016/j.kint.2022.08.026 36108806 PMC9691617

[B19] Grande M. T. Sánchez-Laorden B. López-Blau C. De Frutos C. A. Boutet A. Arévalo M. (2015). Snail1-induced partial epithelial-to-mesenchymal transition drives renal fibrosis in mice and can be targeted to reverse established disease. Nat. Med. 21, 989–997. 10.1038/nm.3901 26236989

[B20] Gu L. Lin J. Gan Y. Xiao R. (2022). Knockdown of SOX9 alleviates tracheal fibrosis through the Wnt/β-catenin signaling pathway. J. Mol. Med. Berl. 100, 1659–1670. 10.1007/s00109-022-02261-9 36192639

[B21] He W. Dai C. Li Y. Zeng G. Monga S. P. Liu Y. (2009). Wnt/beta-catenin signaling promotes renal interstitial fibrosis. J. Am. Soc. Nephrol. 20, 765–776. 10.1681/ASN.2008060566 19297557 PMC2663839

[B22] He W. Tan R. J. Li Y. Wang D. Nie J. Hou F. F. (2012). Matrix metalloproteinase-7 as a surrogate marker predicts renal wnt/β-catenin activity in CKD. J. Am. Soc. Nephrol. 23, 294–304. 10.1681/ASN.2011050490 22095947 PMC3269179

[B23] Hirohama D. Abedini A. Moon S. Surapaneni A. Dillon S. T. Vassalotti A. (2023). Unbiased human kidney tissue proteomics identifies matrix metalloproteinase 7 as a kidney disease biomarker. J. Am. Soc. Nephrol. 34, 1279–1291. 10.1681/ASN.0000000000000141 37022120 PMC10356165

[B24] Hou J. Shi J. Chen L. Lv Z. Chen X. Cao H. (2018). M2 macrophages promote myofibroblast differentiation of LR-MSCs and are associated with pulmonary fibrogenesis. Cell. Commun. Signal 16, 89. 10.1186/s12964-018-0300-8 30470231 PMC6260991

[B25] Hu L. Ding M. He W. (2022). Emerging therapeutic strategies for attenuating tubular EMT and kidney fibrosis by targeting wnt/β-catenin signaling. Front. Pharmacol. 12, 830340. 10.3389/fphar.2021.830340 35082683 PMC8784548

[B26] Huang R. Fu P. Ma L. (2023). Kidney fibrosis: from mechanisms to therapeutic medicines. Signal Transduct. Target. Ther. 8, 129. 10.1038/s41392-023-01379-7 36932062 PMC10023808

[B27] Jiang Y. Xiang C. Zhong F. Zhang Y. Wang L. Zhao Y. (2021). Histone H3K27 methyltransferase EZH2 and demethylase JMJD3 regulate hepatic stellate cells activation and liver fibrosis. Theranostics 11, 361–378. 10.7150/thno.46360 33391480 PMC7681085

[B28] La Russa A. Serra R. Faga T. Crugliano G. Bonelli A. Coppolino G. (2024). Kidney fibrosis and matrix metalloproteinases (MMPs). Front. Biosci. Landmark Ed. 29, 192. 10.31083/j.fbl2905192 38812325

[B29] Li H. Leung J. C. K. Yiu W. H. Chan L. Y. Y. Li B. Lok S. W. Y. (2022a). Tubular β-catenin alleviates mitochondrial dysfunction and cell death in acute kidney injury. Cell. Death Dis. 13, 1061. 10.1038/s41419-022-05395-3 36539406 PMC9768165

[B30] Li L. Fu H. Liu Y. (2022b). The fibrogenic niche in kidney fibrosis: components and mechanisms. Nat. Rev. Nephrol. 18, 545–557. 10.1038/s41581-022-00590-z 35788561

[B31] Lin S.-L. Li B. Rao S. Yeo E. J. Hudson T. E. Nowlin B. T. (2010). Macrophage Wnt7b is critical for kidney repair and regeneration. Proc. Natl. Acad. Sci. 107, 4194–4199. 10.1073/pnas.0912228107 20160075 PMC2840080

[B32] Little M. H. McMahon A. P. (2012). Mammalian kidney development: principles, progress, and projections. Cold Spring Harb. Perspect. Biol. 4, a008300. 10.1101/cshperspect.a008300 22550230 PMC3331696

[B33] Liu J. (2022). Wnt/β-catenin signalling: function, biological mechanisms, and therapeutic opportunities. Signal Transduct. Target. Ther. 7, 3. 10.1038/s41392-021-00762-6 34980884 PMC8724284

[B34] Liu Y. (2011). Cellular and molecular mechanisms of renal fibrosis. Nat. Rev. Nephrol. 7, 684–696. 10.1038/nrneph.2011.149 22009250 PMC4520424

[B35] Lovisa S. LeBleu V. S. Tampe B. Sugimoto H. Vadnagara K. Carstens J. L. (2015). Epithelial-to-mesenchymal transition induces cell cycle arrest and parenchymal damage in renal fibrosis. Nat. Med. 21, 998–1009. 10.1038/nm.3902 26236991 PMC4587560

[B36] Luo C. Zhou S. Zhou Z. Liu Y. Yang L. Liu J. (2018). Wnt9a promotes renal fibrosis by accelerating cellular senescence in tubular epithelial cells. J. Am. Soc. Nephrol. 29, 1238–1256. 10.1681/ASN.2017050574 29440280 PMC5875944

[B37] Maarouf O. H. Aravamudhan A. Rangarajan D. Kusaba T. Zhang V. Welborn J. (2016). Paracrine Wnt1 drives interstitial fibrosis without inflammation by tubulointerstitial cross-talk. J. Am. Soc. Nephrol. 27, 781–790. 10.1681/ASN.2014121188 26204899 PMC4769196

[B38] Miao Y. Ha A. de Lau W. Yuki K. Santos A. J. M. You C. (2020). Next-Generation surrogate Wnts support organoid growth and deconvolute frizzled pleiotropy *in vivo* . Cell. Stem Cell. 27, 840–851. 10.1016/j.stem.2020.07.020 32818433 PMC7655723

[B39] Morgado-Pascual J. L. Suarez-Alvarez B. Marchant V. Basantes P. Tharaux P. L. Ortiz A. (2022). Type IV collagen and SOX9 are molecular targets of BET inhibition in experimental glomerulosclerosis. Int. J. Mol. Sci. 24, 486. 10.3390/ijms24010486 36613933 PMC9820124

[B40] Murea M. Park J. K. Sharma S. Kato H. Gruenwald A. Niranjan T. (2010). Expression of Notch pathway proteins correlates with albuminuria, glomerulosclerosis, and renal function. Kidney Int. 78, 514–522. 10.1038/ki.2010.172 20531454 PMC3164583

[B41] Nlandu-Khodo S. Osaki Y. Scarfe L. Yang H. Phillips-Mignemi M. Tonello J. (2020). Tubular β-catenin and FoxO3 interactions protect in chronic kidney disease. JCI Insight 5, e135454. 10.1172/jci.insight.135454 32369448 PMC7259539

[B42] Nusse R. Clevers H. (2017). Wnt/β-Catenin signaling, disease, and emerging therapeutic modalities. Cell. 169, 985–999. 10.1016/j.cell.2017.05.016 28575679

[B43] Pai C.-H. Lin S. R. Liu C. H. Pan S. Y. Hsu H. Chen Y. T. (2020). Targeting fibroblast CD248 attenuates CCL17-expressing macrophages and tissue fibrosis. Sci. Rep. 10, 16772. 10.1038/s41598-020-73194-x 33033277 PMC7544830

[B44] Qi R. Wang J. Jiang Y. Qiu Y. Xu M. Rong R. (2021). Snai1-induced partial epithelial-mesenchymal transition orchestrates p53-p21-mediated G2/M arrest in the progression of renal fibrosis via NF-κB-mediated inflammation. Cell. Death Dis. 12, 44. 10.1038/s41419-020-03322-y 33414422 PMC7790819

[B45] Ramakrishna K. Nalla L. V. Naresh D. Venkateswarlu K. Viswanadh M. K. Nalluri B. N. (2023). WNT-Β catenin signaling as a potential therapeutic target for neurodegenerative diseases: current status and future perspective. Diseases 11, 89. 10.3390/diseases11030089 37489441 PMC10366863

[B46] Raza S. Jokl E. Pritchett J. Martin K. Su K. Simpson K. (2021). SOX9 is required for kidney fibrosis and activates NAV3 to drive renal myofibroblast function. Sci. Signal 14, eabb4282. 10.1126/scisignal.abb4282 33653921

[B47] Ruiz-Ortega M. Rayego-Mateos S. Lamas S. Ortiz A. Rodrigues-Diez R. R. (2020). Targeting the progression of chronic kidney disease. Nat. Rev. Nephrol. 16, 269–288. 10.1038/s41581-019-0248-y 32060481

[B48] Saha S. Aranda E. Hayakawa Y. Bhanja P. Atay S. Brodin N. P. (2016). Macrophage-derived extracellular vesicle-packaged WNTs rescue intestinal stem cells and enhance survival after radiation injury. Nat. Commun. 7, 13096. 10.1038/ncomms13096 27734833 PMC5065628

[B49] Satoh M. Nagasu H. Morita Y. Yamaguchi T. P. Kanwar Y. S. Kashihara N. (2012). Klotho protects against mouse renal fibrosis by inhibiting Wnt signaling. Am. J. Physiol-renal. 303, F1641–F1651. 10.1152/ajprenal.00460.2012 PMC3532475 23034937

[B50] Schunk S. J. Floege J. Fliser D. Speer T. (2021). WNT-β-catenin signalling - a versatile player in kidney injury and repair. Nat. Rev. Nephrol. 17, 172–184. 10.1038/s41581-020-00343-w 32989282

[B51] Serafino A. Giovannini D. Rossi S. Cozzolino M. (2020). Targeting the Wnt/β-catenin pathway in neurodegenerative diseases: recent approaches and current challenges. Expert Opin. Drug Discov. 15, 803–822. 10.1080/17460441.2020.1746266 32281421

[B52] Sheng L. Zhuang S. (2020). New insights into the role and mechanism of partial epithelial-mesenchymal transition in kidney fibrosis. Front. Physiol. 11, 569322. 10.3389/fphys.2020.569322 33041867 PMC7522479

[B53] Song S. Huang H. Guan X. Fiesler V. Bhuiyan M. I. H. Liu R. (2021). Activation of endothelial Wnt/β-catenin signaling by protective astrocytes repairs BBB damage in ischemic stroke. Prog. Neurobiol. 199, 101963. 10.1016/j.pneurobio.2020.101963 33249091 PMC7925353

[B54] Su X. Zhou G. Tian M. Wu S. Wang Y. (2021). Silencing of RSPO1 mitigates obesity-related renal fibrosis in mice by deactivating Wnt/β-catenin pathway. Exp. Cell. Res. 405, 112713. 10.1016/j.yexcr.2021.112713 34181940

[B55] Tan T. K. Zheng G. Hsu T. T. Lee S. R. Zhang J. Zhao Y. (2013). Matrix metalloproteinase-9 of tubular and macrophage origin contributes to the pathogenesis of renal fibrosis via macrophage recruitment through osteopontin cleavage. Lab. Invest. 93, 434–449. 10.1038/labinvest.2013.3 23358111

[B56] Tian X.-J. Zhou D. Fu H. Zhang R. Wang X. Huang S. (2020). Sequential Wnt agonist then antagonist treatment accelerates tissue repair and minimizes fibrosis. iScience 23, 101047. 10.1016/j.isci.2020.101047 32339988 PMC7186527

[B57] Tian Y. Chen J. Huang W. Ren Q. Feng J. Liao J. (2024). Myeloid-derived Wnts play an indispensable role in macrophage and fibroblast activation and kidney fibrosis. Int. J. Biol. Sci. 20, 2310–2322. 10.7150/ijbs.94166 38617540 PMC11008274

[B58] Timperi E. Gueguen P. Molgora M. Magagna I. Kieffer Y. Lopez-Lastra S. (2022). Lipid-associated macrophages are induced by cancer-associated fibroblasts and mediate immune suppression in breast cancer. Cancer Res. 82, 3291–3306. 10.1158/0008-5472.CAN-22-1427 35862581

[B59] Trogisch F. A. Abouissa A. Keles M. Birke A. Fuhrmann M. Dittrich G. M. (2024). Endothelial cells drive organ fibrosis in mice by inducing expression of the transcription factor SOX9. Sci. Transl. Med. 16, eabq4581. 10.1126/scitranslmed.abq4581 38416842

[B60] Wang Q. Sun Z.-X. Allgayer H. Yang H.-S. (2010). Downregulation of E-cadherin is an essential event in activating beta-catenin/Tcf-dependent transcription and expression of its target genes in Pdcd4 knockdown cells. Oncogene 29, 128–138. 10.1038/onc.2009.302 19784072 PMC2920641

[B61] Wozniak J. Floege J. Ostendorf T. Ludwig A. (2021). Key metalloproteinase-mediated pathways in the kidney. Nat. Rev. Nephrol. 17, 513–527. 10.1038/s41581-021-00415-5 33879883

[B62] Xiao L. Zhou D. Tan R. J. Fu H. Zhou L. Hou F. F. (2016). Sustained activation of wnt/β-catenin signaling drives AKI to CKD progression. J. Am. Soc. Nephrol. 27, 1727–1740. 10.1681/ASN.2015040449 26453613 PMC4884114

[B63] Xie H. Miao N. Xu D. Zhou Z. Ni J. Yin F. (2021). FoxM1 promotes Wnt/β-catenin pathway activation and renal fibrosis via transcriptionally regulating multi-Wnts expressions. J. Cell. Mol. Med. 25, 1958–1971. 10.1111/jcmm.15948 33434361 PMC7882937

[B64] Yang D. Li Q. Shang R. Yao L. Wu L. Zhang M. (2020). WNT4 secreted by tumor tissues promotes tumor progression in colorectal cancer by activation of the Wnt/β-catenin signalling pathway. J. Exp. Clin. Cancer Res. 39, 251. 10.1186/s13046-020-01774-w 33222684 PMC7682076

[B65] Zhang Y.-L. Tang T. T. Wang B. Wen Y. Feng Y. Yin Q. (2024). Identification of a novel ECM remodeling macrophage subset in AKI to CKD transition by integrative spatial and single-cell analysis. Adv. Sci. (Weinh) 11, e2309752. 10.1002/advs.202309752 39119903 PMC11481374

[B66] Zhou D. Fu H. Xiao L. Mo H. Zhuo H. Tian X. (2018a). Fibroblast-specific β-catenin signaling dictates the outcome of AKI. J. Am. Soc. Nephrol. 29, 1257–1271. 10.1681/ASN.2017080903 29343518 PMC5875957

[B67] Zhou D. Fu H. Zhang L. Zhang K. Min Y. Xiao L. (2017b). Tubule-derived Wnts are required for fibroblast activation and kidney fibrosis. J. Am. Soc. Nephrol. 28, 2322–2336. 10.1681/ASN.2016080902 28336721 PMC5533232

[B68] Zhou D. Li Y. Lin L. Zhou L. Igarashi P. Liu Y. (2012). Tubule-specific ablation of endogenous β-catenin aggravates acute kidney injury in mice. Kidney Int. 82, 537–547. 10.1038/ki.2012.173 22622501 PMC3425732

[B69] Zhou D. Tian Y. Sun L. Zhou L. Xiao L. Tan R. J. (2017a). Matrix metalloproteinase-7 is a urinary biomarker and pathogenic mediator of kidney fibrosis. J. Am. Soc. Nephrol. 28, 598–611. 10.1681/ASN.2016030354 27624489 PMC5280025

[B70] Zhou L. Chen X. Lu M. Wu Q. Yuan Q. Hu C. (2019). Wnt/β-catenin links oxidative stress to podocyte injury and proteinuria. Kidney Int. 95, 830–845. 10.1016/j.kint.2018.10.032 30770219 PMC6431566

[B71] Zhou X. Chen H. Hu Y. Ma X. Li J. Shi Y. (2023). Enhancer of zeste homolog 2 promotes renal fibrosis after acute kidney injury by inducing epithelial-mesenchymal transition and activation of M2 macrophage polarization. Cell. Death Dis. 14, 253. 10.1038/s41419-023-05782-4 37029114 PMC10081989

[B72] Zhou X. Xiong C. Tolbert E. Zhao T. C. Bayliss G. Zhuang S. (2018b). Targeting histone methyltransferase enhancer of zeste homolog-2 inhibits renal epithelial-mesenchymal transition and attenuates renal fibrosis. FASEB J. 32, fj201800237R. 10.1096/fj.201800237R PMC6181636 29775417

[B73] Zhou X. Zang X. Ponnusamy M. Masucci M. V. Tolbert E. Gong R. (2016). Enhancer of zeste homolog 2 inhibition attenuates renal fibrosis by maintaining Smad7 and phosphatase and tensin homolog expression. J. Am. Soc. Nephrol. 27, 2092–2108. 10.1681/ASN.2015040457 26701983 PMC4926973

